# Exhaled patient derived aerosol dispersion during awake tracheal intubation with concurrent high flow nasal therapy

**DOI:** 10.1007/s10877-023-00990-x

**Published:** 2023-03-17

**Authors:** Marc Mac Giolla Eain, Kevin Nolan, Brian Murphy, Conan McCaul, Ronan MacLoughlin

**Affiliations:** 1 Research and Development, Science and Emerging Technologies, Aerogen Ltd, IDA Business Park, Dangan, Galway, H91HE94 Ireland; 2 School of Mechanical and Materials Engineering, University College Dublin, Dublin, Ireland; 3 Department of Anaesthesia, Rotunda Hospital, Parnell Square, Dublin, Ireland; 4 School of Medicine and Medical Science, University College Dublin, Dublin, Ireland; 5 School of Pharmacy and Biomolecular Sciences, Royal College of Surgeons, Dublin, Ireland; 6 School of Pharmacy and Pharmaceutical Sciences, Trinity College, Dublin, Ireland

**Keywords:** Aerosol-generating procedures, COVID-19, Awake tracheal intubation, High flow nasal therapy, Aerosol distribution, SARS-CoV-2

## Abstract

Awake Tracheal Intubation (ATI) can be performed in cases where there is potential for difficult airway management. It is considered an aerosol generating procedure and is a source of concern to healthcare workers due to the risk of transmission of airborne viral infections, such as SARS–CoV-2. At present, there is a lack of data on the quantities, size distributions and spread of aerosol particles generated during such procedures. This was a volunteer observational study which took place in an operating room of a university teaching hospital. Optical particle sizers were used to provide real time aerosol characterisation during a simulated ATI performed with concurrent high-flow nasal oxygen therapy. The particle sizers were positioned at locations that represented the different locations of clinical staff in an operating room during an ATI. The greatest concentration of patient derived aerosol particles was within 0.5–1.0 m of the subject and along their midline, 2242 #/cm^3^. As the distance, both radial and longitudinal, from the subject increased, the concentration decreased towards ambient levels, 36.9 ± 5.1 #/cm^3^. Patient derived aerosol particles < 5 µm in diameter remained entrained in the exhaled aerosol plume and fell to the floor or onto the subject. Patient derived particles > 5 µm in diameter broke away from the exhaled plume and spread radially throughout the operating room. Irrespective of distance and ventilation status, full airborne protective equipment should be worn by all staff when ATI is being performed on patients with suspected viral respiratory infections.

## Introduction

Healthcare workers (HCWs) frequently come into contact with contaminated bodily fluids, surfaces and aerosol-transmitted infections and are, as a consequence, at a constant risk of exposure to a panoply of infectious diseases, with airborne pathogens a particular recent concern in the current COVID-19 pandemic [1]. In prior viral outbreaks of Severe Acute Respiratory Syndrome (SARS) and Middle East Respiratory Syndrome (MERS), despite the use of safety protocols and personal protective equipment (PPE), HCWs accounted for approximately 20% of all cases, with intensive care doctors a particularly high risk group [2]. In the current pandemic, it has been shown that patient facing HCWs are much more likely to acquire infection than those with more remote roles [3].

This risk of disease acquisition has led to improvements in strategies and equipment to minimise transmission, which should be proportionate to risk, which is in turn driven by environmental risk. In addition to vaccination, PPE is the mainstay of HCW protection, but varies considerably in efficacy and it is necessary to understand when higher level protection, which is typically less accessible and more expensive than less effective equipment, is necessary in order to match resources appropriately. Airborne viral infections such as Human Avian Influenza A (H5N1) [4] and MERS [5] are transmitted as respiratory droplets from infected patients [6, 7]. Individuals expel aerosol particles from their noses and mouths while breathing, speaking, laughing, coughing and many other activities [8]. Patients with viral respiratory infections, such as Severe Acute Respiratory Syndrome CoronaVirus (SARS-CoV-2), harbour large viral loads in their respiratory tracts [9, 10]. Of particular concern to HCWs is the potential for disease transmission during ventilatory support for active or imminent respiratory failure as this is frequently provided non-invasively through systems such as high flow nasal oxygen (HFNO). These systems drive oxygen through the nares into the lungs and the exhalate, with contains patient derived bioaerosols, is returned to the immediate environment without filtration. Such oxygenation supplementation systems are also used in the context of other airway interventions such as Awake Tracheal Intubation (ATI), which brings the intubator to within 1 m of the open airway and is the main focus of this investigation. ATI is typically performed facing the patient, who is in a sitting position, sedated and oxygenated with HFNO. ATI can be complicated by coughing, which is likely to generate up to 500,000 aerosol particles [8], ranging in size from 0.01 to 1000 µm in diameter and consists of a cocktail of water, mucus and a number of other, potentially infectious elements [11].

The state-of-the-art knowledge in relation to the size and distribution of potentially infectious bioaerosols during HFNO use is in incomplete. Existing work has examined aerosol generation without detailed determination of direction and extent of distal spread [12]. This work aims to address this gap in the literature. This study focused on the distribution of aerosol particles exhaled by the patient. We wished to assess the pattern of potential aerosol distribution following exhalation, while using HFNO with a view to enhance the understanding of the risk of disease transmission in the healthcare environment, with a particular focus on likely exposure risk for an intubator and support staff during ATI.

## **Methods**

**Fig. 1:**
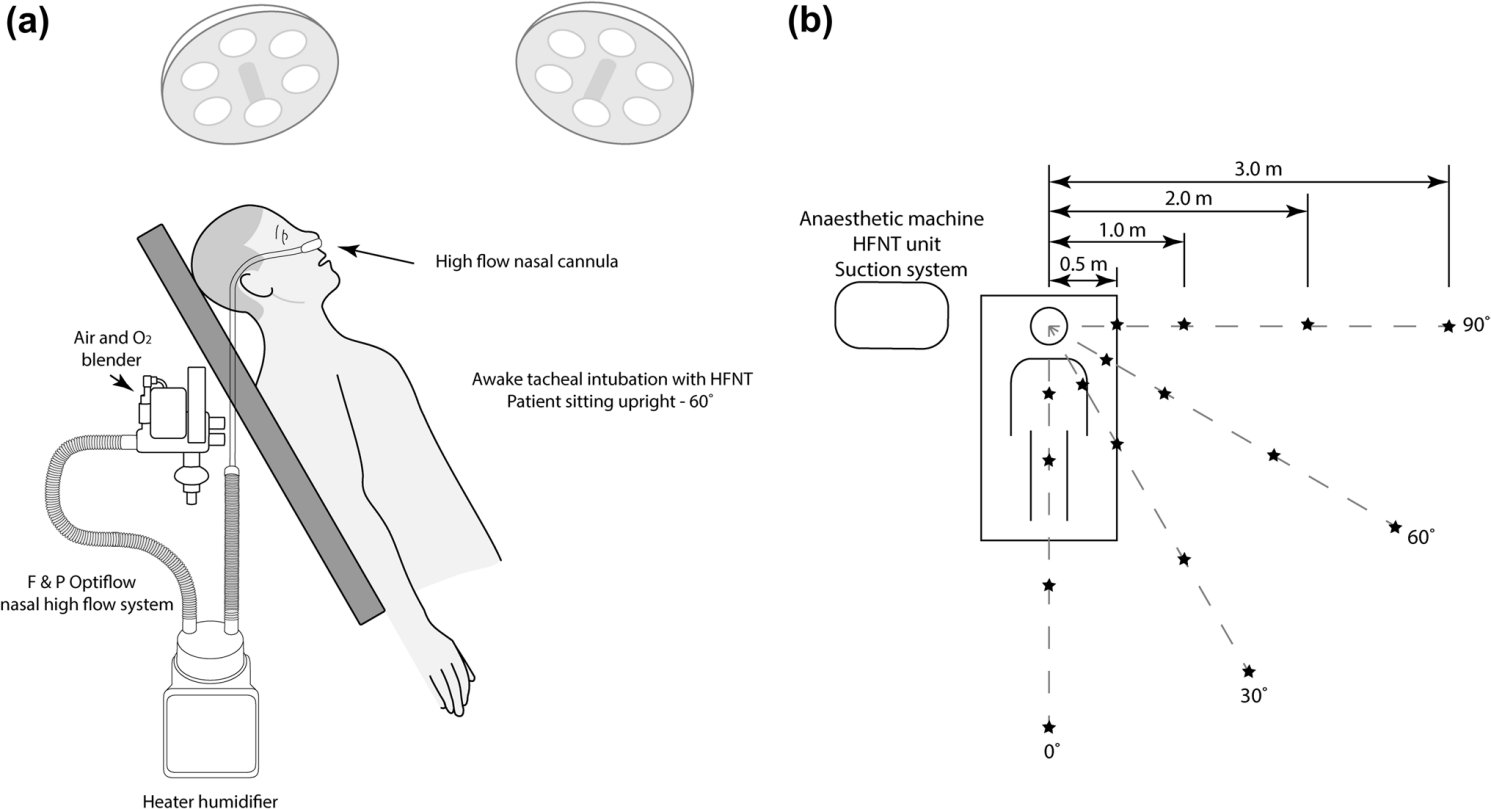
Schematic illustration of the setup used to measure the release and dispersion of exhaled aerosol during Awake Tracheal Intubation (ATI) with concurrent High Flow Nasal Oxygen (HFNO) therapy. The symbol (*) denotes the Optical Particle Sizer (OPS) measurement locations relative to the test subject

This was a single healthy volunteer observational study (male, age: 37, body weight: 84 kg) which took place in an operating room of a university teaching hospital. The study took place following approval by the local ethics committee (Institutional Review Board Number 1/378/2172). Figure 1 is a schematic illustration of the test setup that was used to examine the aerosol dispersion around the volunteer.

The volunteer was seated in a semi-erect position on an anaesthetic trolley, at 60° to the horizontal (a). This position was chosen due its physiological and anatomical benefits for both the patient and practitioner and is the recommended position for a patient during ATI [13–15]. Heated humidified air (MR 850 Optiflow system, F & P Healthcare, New Zealand) was supplied to the subject at flow rates of 10, 30, 50 and 70 Litres per minute (LPM). A simple saline solution (0.9% sodium chloride, Braun, Germany) was nebulised (Aerogen Solo, Aerogen, Ireland) and delivered, via the nasal cannula (OPT + 944, F & P Healthcare, New Zealand).

Two optical particle sizers (OPS) (OPS3300, TSI Inc., USA) were used to characterise the exhaled aerosol flow as previously described in [16, 17]. The OPS operates on the principle of optical scattering from single particles and is fully complaint with ISO 21501-04:2018 Determination of particle size distribution—Single particle light interaction methods [18]. Particles are illuminated using a laser beam shaped to a thin sheath focused below the inlet sampling nozzle port. As the particles pass through this light sheath, they scatter light in the form of pulses. The particle pulse heights are proportional to the optical particle size. The system is calibrated using different monodispersed uniformly shaped polystyrene latex particles, where the different pulse heights are related to the different sized particles. The OPS characterised particles with optical diameters in the range 0.3–10 µm in 16 different bins. The sampling port, 6.35 mm in diameter, is positioned at the top of the instrument and is designed so that aerosol particles can be sampled from the open air, at 1 LPM.

The measurement locations are highlighted in Fig. 1b, where the symbol (*) denotes OPS device placement relative to the test subject. The different measurements positions within the test room allowed for the aerosol distribution and particle concentration (#/cm^3^) at various potential intubator and support staff positions to be recorded. The measurement positions ranged from 0.5 to 3.0 m and 0 to 90° from the subject midline, at a height of 1.65 m from the floor, which was approximately in line with the mouth of the test subject. Measurements were taken at 1 s intervals for a total of 1 min during tidal breathing. Ambient aerosol levels were established prior to testing to allow for comparison with the different test conditions considered in this study. A series of three (3) independent 5—minute tests, with data recorded at 1 s intervals, recorded the ambient aerosol levels within the operating room. Ambient particle number concentration (PNC) (#/cm^3^) was found to be 36.9 ± 5.1 #/cm^3^. All testing was completed in independent triplicate.

The data was analysed and plotted using MATLAB (MathWorks R2021b) and is presented in the form of radar plots. Radar plots are a graphical technique that allow a visual comparison of multiple independent data sets on a two-dimensional plot.

## Results

**Fig. 2:**
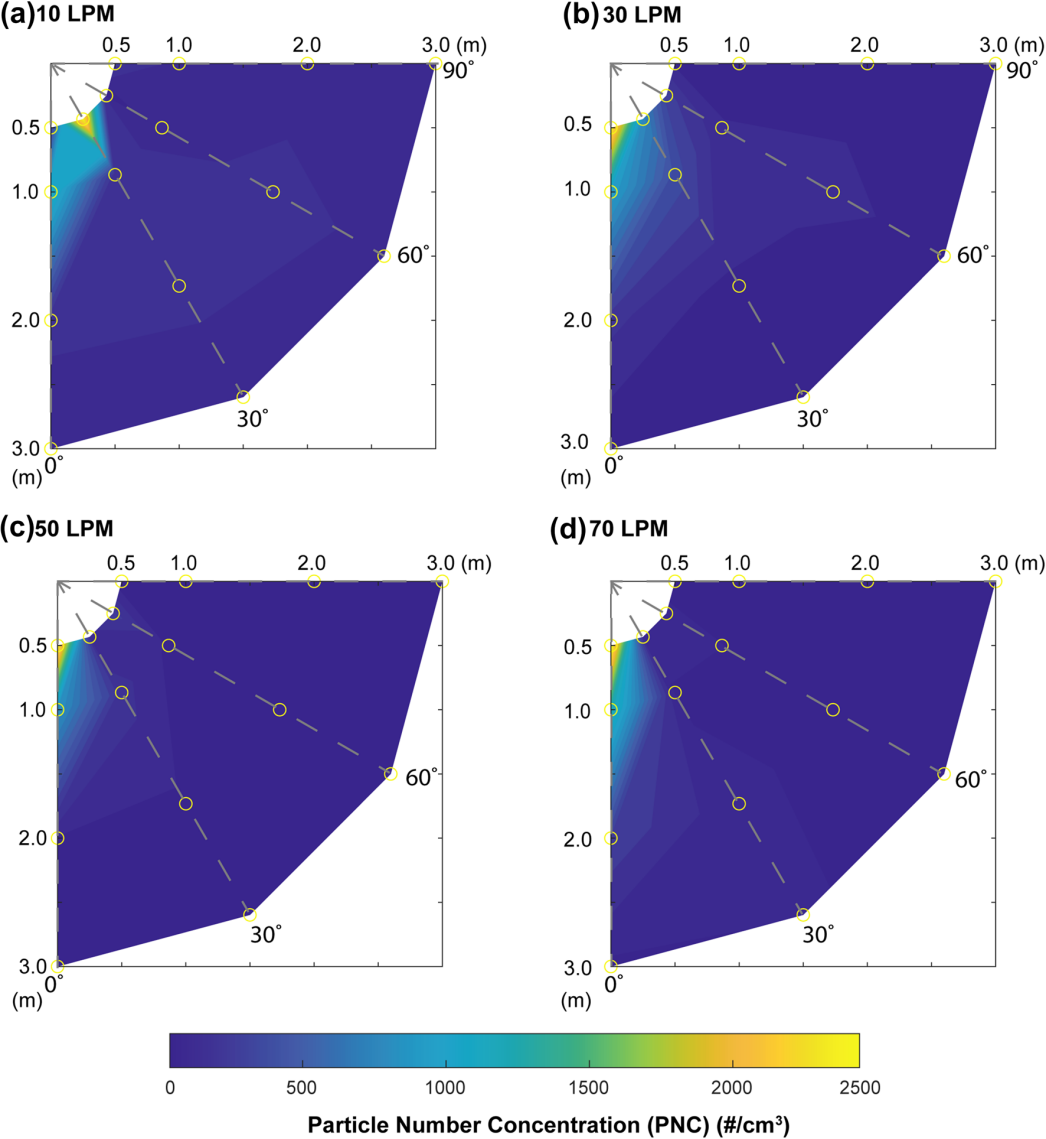
Radar plot illustrating the influence of concurrent high flow oxygen therapy, **a** 10, **b** 30, **c** 50, and **d** 70 Litres per minute (LPM), on the release and distribution of exhaled aerosol particles, in terms of particle number concentration (PNC) (#/cm^3^), during a simulated ATI. The arrow in the figure denotes the direction that the subjects head was facing. The yellow circles denote the OPS measurement positions in relation to the subject

Figure 2 is a radar plot that illustrates the distribution of all the measured exhaled aerosol particles, in terms of particle number concentration (PNC) (#/cm^3^), in the test room at different positions, 0.5–3.0 m and at 0–90° relative to the subject midline, and at different supplemental air flow rates, 10–70 LPM. It can be seen in Fig. 2 that the peak concentration of exhaled aerosol particles is in the area approximately 0.5–1.0 m along the midline of the subject, 0°, particularly at the higher flow rates, 30–70 LPM, the average peak PNC was 2422 #/cm^3^ at 50 LPM. As the distance from the subject increased, 1.0–3.0 m, the PNC decreased towards ambient levels, 36.9 ± 5.1 #/cm^3^. This is particularly evident when moving radially outward from the midline of the subject, 30–90°, irrespective of supplemental gas flow rate.

**Fig. 3:**
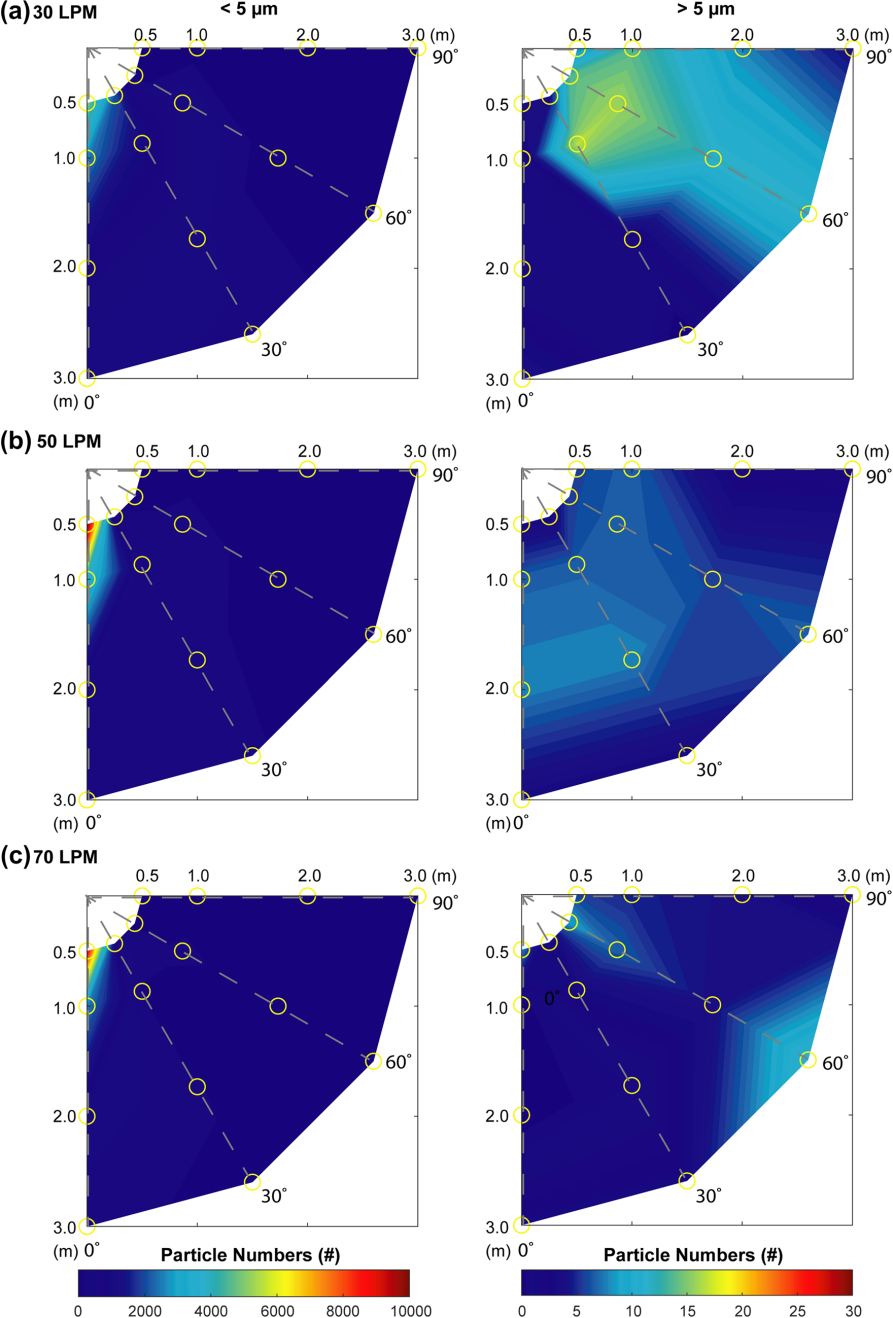
Radar plots that illustrate the distribution of the exhaled aerosol particles above, left column, and below, right column, 5 µm in diameter with **a** 30, **b** 50 and **c** 70 Litres per minute (LPM) of concurrent high flow oxygen therapy. The gold circles indicate the positions of the optical particle sizers relative to the test subject. The data plotted is the average number of exhaled particles (#), n = 3

Figure 3 shows the distribution of exhaled aerosol particles, in terms of relative quantities (#), above and below a threshold of 5 µm in diameter in the operating room at the different positions and supplemental air flow rates. This threshold was chosen as it has been documented in the literature that aerosol particles < 5 µm in diameter carry the highest risk of inhalation and airborne transmission of viral loads [19–21]. The greatest number of potentially respirable particles, < 5 µm, remained entrained in the jet of exhaled aerosol, peak of 9211 particles at 0.5 m and 0° from the subject midline at 50 LPM. The larger diameter particles, > 5 µm, break out from this jet and spread laterally throughout the test room, measured up to 3.0 m from the subject and 30–90° lateral to the midline.

## Discussion

The data from the current study show that during HFNO therapy in the seated position, the distribution pattern of patient generated aerosol varies considerably, with maximal concentration of smaller particles at a distance of 0.5–1.0 m in front of the subject and larger particles dispersed more laterally and distally. This study was designed to strengthen the state-of-the-art knowledge around the risk of environmental exposure for HCWs in close proximity to patients while performing or supporting an ATI. The data suggest that should aerosol be generated during the procedure, HCWs within 3 m of the patient would be exposed to particles in excess of environmental baselines.

Airway management, including intubation and extubation, are considered to be aerosol generating procedures and expose HCWs to increased risk. As a consequence, the Society of Airway Management recommend avoidance of procedures that cause aerosolisaion of secretions [22]. The degree of risk is however uncertain. A recent World Health Organisation (WHO) commissioned report into the risk of aerosolization, dispersion and infection transmission using HFNO in patients with COVID-19 concluded that the risk of airborne transmission was unknown [23]. An expert consensus group has recently declared the issue to be unresolved [24]. However, none of the published work related to the context of procedural risk during ATI.

The broader literature in the area has concentrated on the potential for airway interventions to cause aerosol dispersion and the extent of this dispersion using a range of models and analytical techniques [25]. Non infected subjects do not generate significant aerosol during HFNO in the absence of coughing [21]. However, ATI involves topicalization of the upper airway structures with local anaesthetic solution, with up to 5 mL in common use. This extraneous liquid could become a source for aerosol and requires further evaluation. Furthermore, coughing or violent exhalation is not uncommon during ATI, particularly if topicalization has not been completely effective and would appear to present the greatest risk to the providers as aerosol dispersal has been measured up to 4.4 m from the subject [14, 26].

Additional work has examined the dispersion of smoke particles, using direct visualization of smoke particles < 1 µm in diameter from a manikin and found greater dispersion with higher flow rates of nasal oxygen therapy, with a distance of 17.2 ± 3.3 cm at the highest flow rate of 60 LPM [27]. That study is somewhat limited, and not directly comparable to ours in that the small particle size (1 µm) is not representative of the spectrum of aerosol generated biologically where particles can have a size range of up to 1000 µm. Elshof et al*.* [28] also assessed dispersion of aerosol using a manikin and a lung simulator and assessed smoke particles between 0.3 and 2.5 µm in diameter using passive tracer visualisation and found dispersal range of up to 33.4 cm. These distances are considerably less than our observations. The majority of other studies in the literature that examine the dispersion of aerosol particles during a particular aerosol treatment provide either a 2D visualisation or a single point measurement, [19–21]. The data presented in Figs. 2 and 3 provides a valuable insight into the dispersion of the exhaled aerosol particles in a typical pre-operative or critical care setting. At the lower flow rate, 10 LPM, there is a greater lateral spread of aerosol particles, up to 2 m and 60° from the subject midline. However, as the flow rate increases, the majority of the aerosol particles remain entrained within the exhaled jet along the patient midline, 0°. Thus, health care workers positioned in this region have a greater exposure risk than those positioned around the periphery of a patient.

## Mechanistic explanation

There are several factors that influence the release and dispersion of exhaled aerosol particles in a room. These include, but are not limited to: room size, ventilation, layout, temperature, patient type, interface and procedure [29, 30]. As such, it is necessary to consider these factors when discussing the results of this study. Irrespective of air flow rate, the greatest concentration of particles measured were 0.5–1.0 m along the subject midline, 0°. This is consistent with other similar works that have examined the release and dispersion of aerosol particles during different aerosol therapies. McGrath et al*.* [31] used aerosol particle sizers to measure the release and dispersion of fugitive medical aerosols during HFNO and found that the peak aerosol concentration (mg/m^3^) occurred 0.8 m from the model. Hui et al. [27] demonstrated that an exhalation jet spread up to 1 m from the nostrils of a simulated patient receiving conventional oxygen therapy as the flow rate increased from 1 to 5 LPM. While in a study examining the dispersion distance of aerosol particles from subjects who coughed and were receiving HFNO at 60 LPM, Loh et al. [14] found that particles travelled as far as 4.5 m from the mouth of the subject. The radar plots in Fig. 2 show that the jet of exhaled aerosol particle increases in length as the air flow rate increases, to almost 2 m from the subject at 70 LPM.

It is widely reported in numerous papers and studies that airborne transmission of viral respiratory illnesses is primarily in aerosol particles < 5 µm in diameter [32, 33]. The data plotted in Fig. 3 presents the distribution of aerosol particles in particle numbers (#), above and below this threshold size. The majority of the particles, almost 100%, are < 5 µm in diameter. The small numbers of particles > 5 µm in diameter are the by-product of the normal breathing pattern of the subject and the increased turbulence, deposition and impactive losses in the tubing of the HFNO system [34]. The smaller diameter particles remain entrained in the exhaled jet and there is no lateral spread of aerosol, particularly as the flow rate increases. The greater number of particles and their concentration levels, Fig. 2, would carry the largest viral loads, remain airborne for longer periods and be of greatest risk of inhalation [35, 36]. Respiratory events such as coughing and sneezing generate larger diameter aerosol droplets, > 5 µm [37, 38]. It can be seen in Fig. 3 that particles > 5 µm in diameter break away from the exhaled jet and spread radially through-out the room. This effect is particularly evident at 10–30 LPM, where the greatest number of these larger diameter exhaled aerosol droplets were detected 1–3 m and 30–60° from the subject. It has long been accepted that larger diameter droplets will settle quickly, < 1 m from the source [39]. The data presented in Fig. 3 indicates that this is not necessarily the case, that there is a greater spread of the larger diameter aerosol droplets than previously thought. Furthermore, procedures such as bronchoscopy and laryngoscopy induce coughing, which will generate large volumes of these larger diameter droplets. These larger droplets are more likely to deposit on surfaces within the room and pose a risk of transmission through physical contact [39]. However, there is also a potential inhalation risk, where the droplets can deposit through impaction in the oropharynx and can potentially be swallowed [40].

This work highlights the potential aerosol exposure hot spots within a simulated clinical setting. These hot spots are the region directly along the patient midline, i.e., the end of the bed, for aerosol particles, < 5 µm, and around the periphery of the patient, 30–60° and 1–3 m, for aerosol droplets, > 5 µm. The WHO guidelines for infection prevention and control of acute respiratory infections in healthcare settings recommends that HCWs should wear a surgical mask, eye protection and take contact precautions if within 2 m from a potentially infectious patient [41]. However, this work suggests that HCWs should wear full airborne protective equipment, N95 mask or equivalent, gown, gloves, goggles, hair covers, and face shield or hood, in any enclosed setting, irrespective of distance and ventilation rate. Furthermore, if possible, all aerosol generating procedures or procedures known to generate respiratory events, such as coughing or sneezing, should be performed in negative pressure environments.

While this work provides some novel insights into the dispersion of exhaled aerosol particles and droplets during an ATI of a subject while undergoing simultaneous HFNO in a clinical setting, there are a number of limitations. This study had a single healthy test subject, the addition of a larger sample size would provide more definitive data and illustrate any trends in the dispersion of the exhaled aerosols. A single patient position used during ATI was examined in this study, a comparison with other potential patient and practitioner positions used would help to inform practitioners and administrators on the safest position for clinical staff. The dispersion of aerosol particles is dependent on many parameters, such as temperature, humidity and ventilation rate and different combinations of these parameters. Slight variations in any of these parameters could have a significant effect on the dispersion of exhaled aerosol particles and droplets. However, these were beyond the scope of this work.

## Conclusion

Our data demonstrate the potential spread of exhaled patient generated aerosol in the vicinity of a patient wearing and using HFNO across the ranges of HCW positioning during ATI. It was found that the greatest concentration of exhaled aerosol particles, (#/cm^3^), was within 1.0 m of the subject mouth and directly along their midline. The smaller diameter aerosol particles, < 5 µm, remained entrained within the exhaled jet, while the larger diameter aerosol droplets, > 5 µm, spread radially through-out the operating room. As such, caregivers should don full airborne protective equipment when entering any room where such a procedure is being performed. The novel findings presented in this exploratory work should help to inform administrators, policy setters and practitioners as to the potential exposure risks and appropriate safety measures to implement during an ATI performed concurrent to HFNO.
